# Interventions in Primary Care to Improve Cervical Cancer Screening: A Systematic Review

**DOI:** 10.7759/cureus.99604

**Published:** 2025-12-19

**Authors:** Anas E Ahmed, Wasan M Hatan, Shahad E Alrehaili, Waad R Alghamdi, Haya K Altarif, Ghala S Alotaibi, Heba Y Alkhamis, Refal F Alfaya, Hassan A Mejamemi, Hoor I Aljasas

**Affiliations:** 1 College of Medicine, Jazan University, Jazan, SAU; 2 College of Medicine, Taibah University, Madinah, SAU; 3 College of Medicine, Najran University, Najran, SAU; 4 College of Medicine, Aljouf University, Aljouf, SAU; 5 College of Medicine, King Faisal University, Al Ahsa, SAU; 6 College of Medicine, King Khalid University, Abha, SAU; 7 Faculty of Medicine, University of Oradea, Oradea, ROU; 8 Faculty of Medicine, Alexandria University, Alexandria, EGY

**Keywords:** cervical cancer screening, culturally tailored interventions, digital education tools, family medicine, human papillomavirus self-sampling, multimodal outreach, primary care, screening uptake, short message service reminders, underserved populations

## Abstract

Cervical cancer screening is a vital preventive service commonly delivered within primary care, yet participation remains suboptimal, particularly among underserved or hard-to-reach groups. This systematic review evaluated the effectiveness of interventions implemented in primary care settings to improve screening uptake, knowledge, and related outcomes. A comprehensive search across major databases identified studies assessing interventions designed to increase screening participation within primary care or family medicine. Ten studies met the inclusion criteria, featuring approaches such as mailed and telephone reminders, short message service (SMS) outreach, multimodal contact strategies, digital educational tools, provider-directed prompts, and human papillomavirus (HPV) self-sampling. Reminder-based interventions consistently increased attendance, with telephone and multimodal outreach showing the greatest gains. Digital tools enhanced patient knowledge and communication, while HPV self-sampling yielded the largest improvements in screening uptake, particularly among Indigenous, long-overdue, and other underserved groups. Culturally tailored strategies produced modest but meaningful benefits for marginalized populations. Overall, randomized studies demonstrated acceptable methodological quality, while observational designs showed moderate to serious concerns. The evidence indicates that multimodal reminders, digital education, and HPV self-sampling are effective components of primary care-based efforts to improve cervical cancer screening, and integrating culturally responsive approaches may help address persistent disparities.

## Introduction and background

Cervical cancer remains a significant global health challenge despite being one of the most preventable malignancies [1,2]. Persistent infection with high-risk human papillomavirus (HPV) is the primary cause [3], and well-organized screening programs have substantially reduced disease incidence and mortality [4,5]. However, disparities in access, utilization, and follow-up of screening services continue across many regions [6]. Primary care plays a central role in prevention, as family medicine clinicians provide accessible, continuous care and are well-positioned to identify women overdue for screening and offer culturally sensitive counseling [7]. Yet, barriers such as limited consultation time, fragmented registries, inconsistent reminder systems, and high clinical workload often hinder optimal delivery of preventive services [8].

A range of strategies has been introduced to improve screening participation within primary care, including mailed letters, short message service (SMS) reminders, telephone outreach, electronic patient portal alerts, and in-clinic educational tools [1,9]. Digital platforms have further expanded opportunities for automated reminders and patient engagement [2,3]. HPV self-sampling has emerged as an important alternative for women facing psychological, cultural, or logistical barriers to clinic-based testing, and evidence suggests that it can substantially increase participation among those overdue for screening, particularly in underserved populations [4,6].

Despite the availability of these tools, screening disparities persist among immigrant women, socioeconomically disadvantaged groups, and rural populations, driven by factors such as stigma, fear, limited health literacy, and logistical challenges [7-9]. Culturally tailored outreach and patient-centered communication in primary care have shown promise in addressing these barriers [10]. The effectiveness of interventions, however, varies widely depending on population characteristics, implementation quality, and healthcare system context [1-3]. Multimodal outreach strategies generally produce stronger gains than single-modality approaches [4,5], while interventions limited to opportunistic reminders during clinic visits often yield only modest improvements [6]. This systematic review, therefore, examines interventions delivered within primary care that aim to improve cervical cancer screening uptake, inform evidence-based strategies to enhance participation, and reduce persistent disparities.

## Review

Methods

Literature Search Strategy

This systematic review was conducted in accordance with the Preferred Reporting Items for Systematic Reviews and Meta-Analyses (PRISMA) 2020 guidelines [11]. It followed a structured and comprehensive search strategy across PubMed, Scopus, Web of Science, and the Cochrane Library from inception through December 2025. The search combined controlled vocabulary and free-text terms related to cervical cancer screening, including Papanicolaou (Pap) testing, HPV testing, and self-sampling, with terms describing primary care settings such as family medicine, general practice, and community health centers. Search terms also captured intervention modalities such as reminders, invitations, digital outreach, education, mailed kits, and self-sampling. Strategies were adapted for each database to ensure conceptual consistency. Filters restricted results to human subjects and original research. Reviews, commentaries, protocols without findings, and studies unrelated to cervical cancer screening or primary care interventions were excluded.

Eligibility Criteria

Eligibility criteria were defined using a modified PICOS framework (Population, Intervention, Comparator, Outcome, Study design) [12]. Studies were included if they involved women or individuals with a cervix who were due for screening and were receiving care within primary care or family medicine settings. Interventions needed to aim explicitly at increasing screening participation and could include mailed reminders, letters, telephone or SMS outreach, digital or video-based education, clinician prompts, or HPV self-sampling integrated into primary care workflows. Studies were required to have a comparator group and report measurable outcomes such as screening uptake, appointment attendance, HPV test completion, or follow-up indicators. Exclusion criteria included studies not conducted in primary care, interventions without a participation outcome, studies limited to knowledge or attitudes, papers without original results, protocols, reviews, and populations not eligible for screening. No geographic or publication year restrictions were applied.

Study Selection

All retrieved records were imported into a reference manager, and duplicates were removed. Study selection occurred in two stages, conducted independently by two reviewers. First, titles and abstracts were screened to exclude studies outside the scope of cervical cancer screening, primary care settings, or participation-focused interventions. Full texts of potentially eligible studies were then assessed using the same dual-reviewer approach. Discrepancies were resolved through discussion and, when necessary, by a third reviewer. This process ensured transparent, criteria-based inclusion of studies with extractable intervention outcomes.

Data Extraction and Quality Appraisal

Two reviewers independently extracted data using a standardized form. Extracted information included study design, setting, participant characteristics, intervention and comparator details, outcomes, follow-up duration, and numerical effect estimates. Additional information on how interventions were integrated into primary care workflows and reported limitations was also recorded. Quality assessment for randomized controlled trials (RCTs) was conducted using the ROB-2 tool, evaluating potential bias in randomization, deviations from intended interventions, missing outcome data, outcome measurement, and selective reporting [13]. Non-randomized studies were appraised using the ROBINS-I tool, which evaluates confounding, participant selection, intervention classification, deviations from protocol, missing data, and outcome measurement [14]. These assessments informed the interpretation and strength of the synthesized findings.

Results

Study Selection

**Figure 1:**
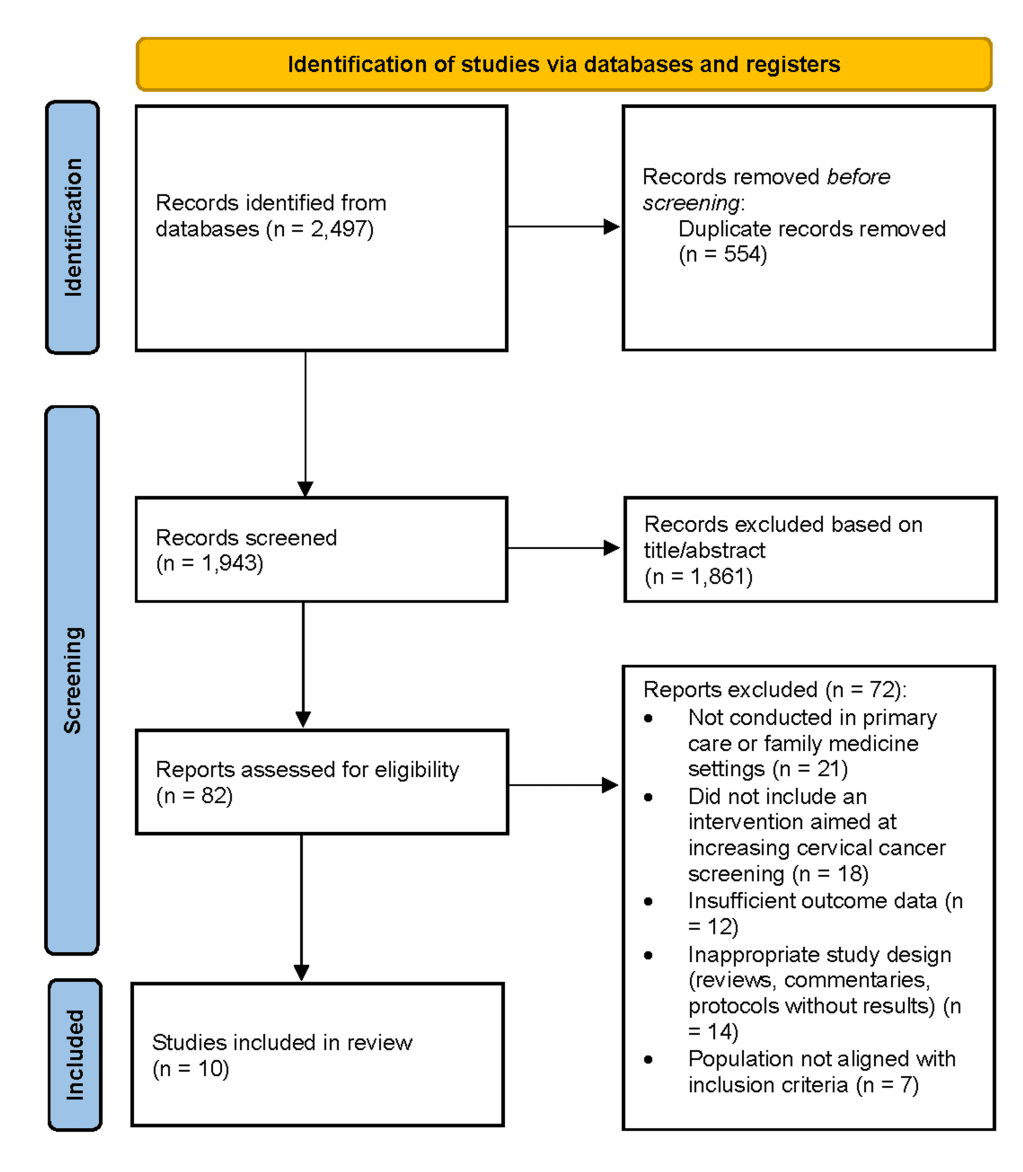
PRISMA flow diagram illustrating the study selection process PRISMA flow diagram illustrating the study selection process for the review [11]. The figure outlines the number of records identified through database searching and other sources, the number of records screened after duplicates were removed, the full-text articles assessed for eligibility, and the final number of studies included in the qualitative and quantitative synthesis, along with reasons for exclusion at each stage.

A total of 2,497 records were identified across all databases. After removing duplicates, 1,943 unique studies were screened, of which 1,861 were excluded based on title and abstract due to irrelevance to primary care, lack of an intervention component, or unsuitable study design. Eighty-two full texts were assessed, and 72 were excluded for reasons including non-primary care settings, absence of a participation-focused intervention, insufficient outcome data, ineligible study designs, or populations outside screening eligibility. Ten studies met all inclusion criteria and were incorporated into the final synthesis and risk-of-bias assessment [1-10] (Figure 1).

Study Characteristics

The included studies were conducted across primary care and family medicine settings in Europe, Oceania, and North America. Most were RCTs, while others used observational or pre-post designs. Populations consisted of adult women eligible for cervical cancer screening, with several studies specifically targeting underserved, Indigenous, rural, or immigrant groups. Interventions ranged from mailed letters, telephone outreach, SMS reminders, and digital education tools to HPV self-sampling and multimodal reminder strategies. Comparators generally included usual care or standard reminder procedures. All studies reported primary outcomes related to screening participation, while secondary outcomes varied and included patient knowledge, communication, follow-up adherence, and economic evaluation. Follow-up durations ranged from single encounters to 18 months. Studies acknowledged limitations such as selection bias, incomplete contact information, short follow-up periods, and implementation challenges in vulnerable populations (Table 1).

**Table 1:** Characteristics of studies included in the systematic review This table summarizes primary care–based interventions to increase cervical cancer screening across multiple countries. All interventions were conducted in primary care or family medicine settings, with outcomes including cervical cancer screening uptake, follow-up compliance, knowledge, and cost-effectiveness measures, as reported in each study. ACCHO = Aboriginal Community Controlled Health Organisation; AOR = adjusted odds ratio; AUD, Australian dollars; cRCT = cluster randomized controlled trial; CI = confidence interval; CRC = colorectal cancer; CST = cervical screening test; EHR = electronic health record; EPR = electronic patient record; EU/EEA, European Union/European Economic Area; GP = general practitioner; HPV = human papillomavirus; ICER = incremental cost-effectiveness ratio; NHS = National Health Service; OR = odds ratio; PHCC = primary health care center; RR = relative risk; SMS = short message service; UCSF = University of California, San Francisco; USA = United States of America

Study (author, year)	Country and setting (primary care/family medicine)	Study design	Population details (sample size, characteristics, inclusion and exclusion criteria)	Intervention description	Comparator/control	Primary outcomes	Secondary outcomes	Follow-up duration	Key results (numerical: OR, RR, %, CI, p-values)	Primary care integration	Study limitations (as reported)
Haith et al. [1]	United Kingdom; rural primary care practice (Marsh Medical Practice, Louth)	Retrospective observational study	N = 1,441 eligible women aged 25–64 years. Age groups: 25–49 (n=680), 50–64 (n=761). Overdue for cervical screening (≥3 years for 25–49, ≥5 years for 50–64). Identified via EPR. Rural setting with high deprivation index. No explicit exclusion criteria besides ineligibility for screening.	Multidisciplinary team–led digital outreach strategy: (1) Educational videos via SMS and email, (2) Informational leaflets, (3) Online booking system, (4) Automated patient identification through EPR, (5) Follow-up messages reinforcing engagement. Content tailored to local population. Implemented using practice software (AccuRx).	No formal control group; pre–post comparison against baseline	Change in cervical screening uptake among eligible women	Qualitative themes describing barriers among persistent non-attenders; subgroup differences by age	3 months	Overall screening completion among overdue patients: 46%. Ages 25–49: 77% → 80.5%. Ages 50–64: 82% → 97%. National target ≥80% achieved. Persistent non-attenders (n=156) described fear, embarrassment, misconceptions, logistical issues. No inferential statistics performed.	Screening outreach entirely performed by primary care practice, integrated into routine workflow; staff-developed materials, automated searches, online booking system; demonstrates scalable model for digital outreach within GP practices	Single practice → limited generalizability; no control group → potential confounding; short follow-up (3 months); retrospective design; limited qualitative response rate (34/156); external public health influences cannot be excluded
Ivers et al. [2]	Australia; ACCHO, New South Wales	Randomized controlled trial	N = 256 women aged 25–74 due for cervical screening. Inclusion: female clients aged 25–74, regular attendees in past 3 years, eligible for screening. Exclusion: hysterectomy, recent abnormal CST, no screening eligibility.	Phone call + up to 2 follow-up SMS reminders. Calls by ACCHO staff; SMS sent if no response. Messages encouraged booking and attendance. Additional education on self-collection for eligible women (≥30 years, ≥2 years overdue, declined speculum exam).	Reminder letters (initial + up to 2 mailed letters)	Completion of CST within 3 months after first reminder	Abnormal CST findings; HPV positivity; response timing; consumer feedback; cost and time spent	3 months	Screening attendance: 17.6% (phone/SMS) vs 12.5% (letter), difference not statistically significant (p = 0.252). Phone/SMS yielded more immediate bookings, but more no-shows. Costs: letter AUD 1.17; SMS/phone ~AUD 0.17.	Fully embedded in primary care: reminders sent through ACCHO staff, use of EMR, follow-up of abnormal results, culturally appropriate care	Small sample; single ACCHO site; low overall response rates; high mobility; inconsistent contact details; non-contactable subgroups; reminder fatigue after 3 attempts
Møen et al. [3]	Norway; 73 general practices in 20 subdistricts of Bergen	Cluster randomized clinical trial	N = 10,360 immigrant women, mean age 44 years, from multiple regions (EU/EEA, Africa, Asia incl. Turkey). Inclusion: immigrant women registered in Norway (2012–2018), assigned to participating GP, aged 25–69. Exclusion: none explicitly stated.	3-component GP-targeted intervention: (1) brief GP educational session on CCS in immigrants, (2) mouse-pad reminder for GPs, (3) multilingual posters in waiting rooms encouraging CCS discussions. GPs instructed to ask every immigrant woman about CCS.	Usual care	Participation in cervical cancer screening (registry) by January 1, 2018	Subgroup analyses by baseline screening status, region/country of origin	~1 year	Screening increased 2.6% in intervention vs 0.6% control. Adjusted OR 1.24 (95% CI 1.11–1.38). Strongest effect in previously unscreened women: OR 1.35 (95% CI 1.16–1.56). Increased screening especially among women from Poland, Pakistan, and Somalia: OR 1.74 (95% CI 1.17–2.61).	Intervention embedded in primary care workflow: GP education, reminders, in-clinic prompts to initiate screening discussions	Absolute effect modest; potential contamination; no GP blinding; registry data may contain misclassification; intervention intensity relatively low
Peitzmeier et al. [4]	USA; urban federally qualified health center (Fenway Health, Boston)	Randomized controlled trial (5 arms)	N = 1,025 eligible patients (initially 1,100 randomized; 75 later found ineligible). Mostly women and transgender men aged 21–64; diverse sexual orientation; 88% insured; all overdue for Pap test. Inclusion: cervical cancer screening overdue, presence of cervix, HIV-negative. Exclusion: HIV-positive, no cervix, prior cervical cancer diagnosis, up-to-date screening.	Four outreach modalities over 3 repeated outreach waves: (1) Letter with educational flyer, (2) Email via secure portal, (3) Telephone calls with scheduling, (4) Multimodal (letter → email → phone). Each is attempted up to 3 times if unscheduled.	Usual care: Pap screening during routine visits	Receipt of Pap test at any time during follow-up	Time to screening; subgroup effects by age; adjusted ORs; proportion receiving at least one outreach attempt	Up to 18 months (523 days)	Pap receipt: 21.4% (control), 29.2% (telephone; AOR=1.7, 95% CI 1.1–2.8), 36.1% (multimodal; AOR=2.3, 95% CI 1.4–3.6). Letter and email are not significantly better than control. Median time to screening: control 270 days, letter 157, email 198, telephone 119, multimodal 122.	Outreach within the primary care center using EHR contact data and communication workflows	Not all patients could be contacted due to missing/incorrect contact info; outcome misclassification risk; lack of blinding; some demographic groups were underpowered; email portal low engagement; real-world contact feasibility varied by modality
López-Torres Hidalgo et al. [5]	Spain; primary care health centers (8 basic health zones in Albacete Health Area)	Randomized experimental study with four parallel arms (written, telephone, face-to-face, control)	N = 1,676 randomized; 1,122 interviewed. Women aged 25–70 years, predominantly White (92.2%), insured under the Spanish NHS. Inclusion: women aged 25–70 assigned to selected primary care zones. Exclusion: history of cervical cancer, hysterectomy, severe sensory or cognitive impairment preventing participation.	Three outreach modalities: (1) written information letter, (2) telephone briefing by health personnel, (3) face-to-face invitation to group educational meeting. All interventions provided structured information on screening recommendations and encouraged attending the health center for Pap testing.	No information provided (“usual care” – no briefing)	Participation in cervical cancer screening within 2 years preceding the interview	Lifetime participation in screening: associations with demographics, perceived health, and cancer worry	2 years	Participation rates: written 35.3% (95% CI 29.8–40.9), telephone 38.4% (95% CI 32.5–44.2), face-to-face 29.3% (95% CI 22.8–35.7), control 26.1% (95% CI 21.2–30.9); p = 0.005. logistic regression: written OR 1.586 (95% CI 1.127–2.233; p = 0.008), telephone OR 1.815 (95% CI 1.286–2.562; p = 0.001), face-to-face not significant. Younger age was associated with higher participation (OR 0.987 per year; p = 0.022).	Primary care clinicians executed all interventions and follow-up within community health centers, demonstrating the feasibility of primary care-based screening recruitment strategies using simple outreach methods.	Low attendance in face-to-face group (31%); potential contamination across groups; variation in provider motivation/communication style; the opportunistic screening context may limit generalizability
Thiel de Bocanegra et al. [6]	USA; 14 primary care clinics (family PACT clinics, California)	Cross-sectional evaluation nested within cluster RCT	N = 229 women, aged 19–35, English/Spanish speaking, diverse ethnic background (majority Latina), low-income population using publicly funded services. Inclusion: women attending participating clinics, aged 19–35, able to complete the survey. Exclusion: none beyond inability to participate.	Tablet-based bilingual cervical cancer education tool delivered before clinical encounter, covering HPV, screening intervals, Pap/HPV follow-up, and communication prompts	Usual care (standard clinic visit)	Knowledge about cervical cancer and HPV; understanding of screening intervals; perceived understanding of abnormal Pap follow-up	Comfort communicating with providers; perceived helpfulness of the tool	Single clinical encounter (immediate, pre-, and post-measurement)	Significantly higher HPV knowledge (p = 0.004) and improved understanding of screening guidelines (p < 0.001). Non-significant trend toward increased comfort communicating with clinicians (p = 0.053). 61.5% reported improved understanding of abnormal Pap management.	Fully embedded in primary care workflow: tool delivered in waiting room before visit to enhance patient–provider communication.	Cross-sectional design prevents causal inference; convenience sampling; self-reported outcomes; short-term assessment; lacks data on actual screening uptake; generalizability limited to similar safety-net clinics
Walsh et al. [7]	USA; 6 diverse primary care clinics (UCSF Clinical Research Network)	Randomized controlled trial	N = 508 adults (251 intervention, 257 control); age 50–70; ~60% women; ethnically diverse (African American, Asian, Hispanic); wide educational range. Inclusion: adults 50–70 with upcoming primary care visits. Exclusion: dementia, severe psychiatric/medical issues, contraindications to screening, or the clinician deemed participation inappropriate.	PreView: interactive, tailored “Video Doctor” via iPad before medical visit. Assessed screening readiness/barriers for breast, cervical, CRC, and prostate screening, provided personalized counseling, and generated a Provider Alert summarizing required screenings.	Video about general healthy lifestyle (no tailored screening content); standard clinical care	Up-to-date completion of: mammography (women 50–70), Pap test (women ≤65), CRC screening (FIT last year or colonoscopy last 10 years), PSA discussion (men)	Screening discussions initiated during visit; improvements in readiness; patient-provider communication	14 months	No significant increases in mammography or Pap test completion. Small non-significant increase in CRC screening (18% vs 12%). Significant increase in PSA discussion: 58% vs 36% (p < 0.01). Intervention increased discussions about mammography, cervical cancer screening, and CRC screening.	Integrated into clinic workflow: patients completed PreView in the waiting room; Provider Alerts were given to clinicians before the encounter to guide preventive care discussions	High baseline screening rates limited the effect size; selection bias (clinicians could exclude patients); self-report bias; limited effect on actual screening completion; study conducted in clinics with strong existing screening performance, limiting generalizability
MacDonald et al. [8]	New Zealand; Six primary care clinics (rural Northland region), partnering with the District Health Board	cRCT	Total recruited Māori women: 931 (intervention 500; control 431). Inclusion: women aged 25–69, last cervical smear ≥4 years ago, registered at participating clinics. Exclusion: age <25 or >69; last smear <4 years; not registered at clinic; hysterectomy. Population largely rural, high deprivation index (76% in the most deprived quintile intervention group).	HPV self-testing is offered through primary care. Women taught by nurses/kaiāwhina to self-collect vaginal HPV samples at clinic, home, or community center; option of clinician-collected HPV test or Pap smear. Outreach by nurses and Māori health workers; free testing; direct referral to colposcopy for HPV16/18/other high-risk types.	Usual care: invitation for clinic-based cervical smear; outreach also available	Cervical screening completion (HPV test or smear), comparing intervention vs control among Māori women	Proportion HPV-positive; follow-up colposcopy attendance; abnormal histology; CIN2/3 detection; screening uptake by time since last screen (>10 years, never screened)	Recruitment March 2018–August 2019; outcome assessment ≥12 months	Screening uptake: 59.0% (295/500) intervention vs 21.8% (94/431) control. Adjusted RR = 2.8 (95% CI 2.4–3.1; p < 0.0001). Not screened ≥10 years: 48% intervention vs 10% control. Never-screened: 42.7% vs 12.7%. HPV positivity: 11% (28/254). Colposcopy attendance: 78.6% (22/28). CIN2+ was detected in 5 women.	Fully embedded in primary care: clinics identified patients, supervised self-testing, coordinated results, and ensured follow-up. Māori health workers are integrated into workflows.	Groups differed at baseline (age, deprivation); not blinded; possible missing eligible patients; some women offered test despite ineligibility; small colposcopy numbers; intention-to-treat analysis; potential contamination unavoidable
Firmino-Machado et al. [9]	Portugal: 13 primary care units within the NHS	Randomized controlled trial (1:1 allocation)	N = 1,220 women, aged 25–49, overdue for cervical screening. Inclusion: women aged 25–49, eligible, overdue, contactable by mobile phone. Exclusion: recent screening, no/incorrect phone number, ineligible for screening.	Automated SMS messages, automated phone calls, and reminder SMS encouraging attendance for the Pap test. Customizable messages delivered through primary care digital systems.	Standard mailed letter invitation (national screening program)	Attendance for cervical cancer screening within 45 days	Screening attendance using an SMS-only pathway	45 days	Intervention attendance: 39.0% vs 25.7% control (p < 0.001). Absolute difference: 13.3% (95% CI 8.1–18.5). SMS-only strategy: –0.4% (not significant).	Fully integrated into primary care workflows; invitations sent by primary care units using clinic systems; appointment booking and screening occurred at GP practices.	Limited to women with mobile phones; short follow-up; SMS-only effectiveness unclear; potential information contamination; no long-term outcomes measured
Paulauskiene et al. [10]	Lithuania; PHCC at Lithuanian University of Health Sciences, Kauno Klinikos	Randomized controlled trial + cost-effectiveness analysis (decision tree model)	Eligible population = 3,294 women aged 25–60 registered at PHCC with no Pap smear in ≥3 years. Randomized by family doctor: Experimental 1,591; Control 1,703. Inclusion: women 25–60, registered, overdue ≥3 years. Exclusion: none explicitly beyond screening ineligibility.	Invitation strategy 2: personal postal invitation letter with pre-booked date/time/place for Pap test. Strategy 3: invitation letter + reminder letter after 1 year if still unscreened. Administrative support from the screening department; email/phone follow-up for results.	Strategy 1: opportunistic screening—verbal invitation by family doctor during routine visits	Screening participation rate and cost per screened woman	Cost per abnormal Pap smear detected; distribution of cytology results; sensitivity analysis	1 year	Participation: after invitation letter 24.6%; after reminder letter +16.9% → 35.6% total. Control: 12.4%. Cytology abnormalities: 25.8% after letter; 22.2% after reminder; 23.7% control. Cost-effectiveness: ICER Strategy 2 vs 1 = €9.67 (per additional screened woman), €55.21 (per additional abnormal Pap detected). Strategy 3 vs 2 = €13.47 and €86.88, respectively.	Screening invitations managed entirely within primary care; letters generated through the PHCC IT system; family doctors’ lists used to identify non-attenders; Pap tests taken within the PHCC.	Conducted in one PHCC only; only direct healthcare costs included; intermediate outcomes used; costs may differ nationally; opportunistic screening outside the study not captured; reminder costs discounted.

Quality Assessment

Among the seven RCTs, most domains were rated as having “some concerns,” reflecting pragmatic implementation in real-world primary care environments. Randomization procedures were generally adequate, and missing outcome data posed minimal risk. Concerns most often arose from deviations from intended interventions, limited blinding, and selective reporting. Overall, each trial was judged as having an acceptable level of methodological rigor. The three non-randomized studies showed greater variability; two were rated as having serious overall risk of bias, and one as moderate. Key limitations included confounding and participant selection, particularly in observational designs. Missing data and outcome measurement concerns were present but generally moderate. Reporting quality was acceptable across all studies (Table 2).

**Table 2:** Quality assessment of included studies This table summarizes risk-of-bias assessments for included studies using the ROB-2 tool [13] for randomized trials and the ROBINS-I tool [14] for non-randomized studies. Risk levels are reported per domain as “low risk,” “some concerns,” “moderate,” or “serious,” indicating increasing likelihood of bias. For ROB-2, domains include randomization process, deviations from intended interventions, missing outcome data, measurement of outcome, and selection of reported results. For ROBINS-I, domains include confounding, selection of participants, classification of interventions, deviations from intended interventions, missing data, measurement of outcomes, and selection of reported results. The overall ROB judgment reflects the cumulative risk across domains for each study. ROB-2 = Risk of Bias 2 tool; ROBINS-I = Risk Of Bias In Non-randomized Studies - of Interventions

Study (author)	Study type/notes	Randomization/confounding	Deviations from intended interventions	Missing outcome data/missing data	Measurement of outcome/measurement of outcomes	Selection of reported results	Overall ROB judgment
Haith et al. (Digital strategies) [1]	ROBINS-I	Serious	Low	Moderate	Moderate	Moderate	Moderate
Ivers et al. [2]	ROB-2	Some concerns	Some concerns	Low risk	Low risk	Some concerns	Some concerns
Møen et al. [3]	ROB-2	Some concerns	Low risk	Low risk	Low risk	Low risk	Some concerns
Peitzmeier et al. [4]	ROB-2	Some concerns	Some concerns	Low risk	Low risk	Some concerns	Some concerns
López-Torres Hidalgo et al. [5]	ROB-2	Low risk	Some concerns	Low risk	Some concerns	Some concerns	Some concerns
Thiel de Bocanegra et al. (Educational tool) [6]	ROBINS-I	Serious	Low	Low	Moderate	Low	Low
Walsh et al. (PreView Trial) [7]	ROB-2	Some concerns	Some concerns	Low risk	Low risk	Some concerns	Some concerns
MacDonald et al. [8]	ROB-2	Low risk	Some concerns	Low risk	Low risk	Some concerns	Some concerns
Firmino-Machado et al. (SCAN) [9]	ROB-2	Low risk	Some concerns	Low risk	Low risk	Some concerns	Some concerns
Paulauskiene et al. (Lithuania invitation system) [10]	ROBINS-I	Moderate	Low	Moderate	Low	Low	Low

Qualitative Synthesis

Across the 10 studies, most interventions demonstrated positive effects on screening participation, although the impact varied across populations and delivery methods. Reminder-based interventions - including letters, telephone calls, and SMS messages - consistently improved screening uptake, with telephone and multimodal outreach showing the strongest gains [2-5,9,10]. Effects were smaller in communities facing substantial structural barriers, such as Aboriginal Australian women, where reminders alone yielded modest improvements [2].

Digital educational tools enhanced patient knowledge, communication, and understanding of HPV and abnormal result management, although effects on actual screening completion varied; some studies showed increases in provider-patient discussions without corresponding behavior change [1,6,7].

HPV self-sampling produced the largest improvements, particularly among Indigenous Māori women, with marked increases in participation compared with usual care and high adherence to follow-up among those with positive results [8].

Studies targeting immigrant or underserved populations showed that culturally tailored outreach, enhanced autonomy, and alternative screening options yielded stronger gains than standard reminder systems. Interventions in these groups highlighted persistent challenges such as stigma, fear, low health literacy, and social vulnerability, which limited the impact of simple communication-based strategies [2,3,8]. Beyond uptake, several studies reported improvements in patient knowledge, communication quality, and cost-effectiveness, indicating broader benefits of interventions that support navigation and engagement within primary care [6,7,10].

## Conclusions

Primary care-based interventions play a pivotal role in improving cervical cancer screening uptake. Reminder systems, particularly telephone and multimodal outreach, consistently increase participation across varied patient groups. HPV self-sampling demonstrates the strongest impact, especially in underserved communities where traditional screening barriers are more prominent. Digital educational tools further support patient understanding and informed decision-making. Overall, primary care remains a key driver for expanding screening coverage and reducing disparities in access.
